# Derivation of a pediatric growth curve for inferior vena caval diameter in healthy pediatric patients: brief report of initial curve development

**DOI:** 10.1186/2036-7902-4-12

**Published:** 2012-05-28

**Authors:** Elizabeth J Haines, Gerardo C Chiricolo, Kresimir Aralica, William M Briggs, Robert Van Amerongen, Andrew Laudenbach, Kevin O’Rourke, Lawrence Melniker

**Affiliations:** 1 Department of Emergency Medicine, New York Methodist Hospital, 506 6th Street, Brooklyn, NY, 11215, USA; 2 Department of Pediatrics, New York Methodist Hospital, 506 6th Street, Brooklyn, NY, 11215, USA

## Abstract

**Background:**

A validated tool has long been sought to provide clinicians with a uniform and accurate method to assess hydration status in the pediatric emergency medicine population. Outpatient clinicians use CDC height- and weight-based curves for the assessment of physical development. In hospital, daily weights provide objective data; however, these are usually not available at presentation.

One of the most promising techniques for the rapid assessment of volume is ultrasound (US) to obtain an indexed inferior vena cava diameter (IVCDi); as previously described. Prior studies have focused on IVCDi in dehydrated patients and have shown that it provides accurate estimates of right atrial pressure and volume status. The objective of this study is to derive an IVC growth curve in healthy pediatric patients.

**Methods:**

Prospective cohort design enrolled healthy children between the ages of 4 weeks and 20 years. Patients presenting with fever, illnesses, or diagnoses known to affect the volume will be excluded. All eligible patients under 21, who have provided self or parental written consent, will undergo a brief ultrasound to obtain transverse and long images of both the IVC and the aorta; all scans will be digitally saved. Image quality will be subjectively rated as poor, fair, or good based on wall clarity. Poor quality images will be recorded but may be omitted from our analysis. Five clinicians completed a 1-h introduction to IVC-US and ten supervised scans prior to enrollment. Still images will be measured in order to determine IVCDi in both transverse and longitudinal planes. To assess inter-rater reliability, in 10% of cases, two clinicians will complete scans. All study scans will be over-read by a fellowship-trained sonologist.

IVCDi will be plotted independently as functions of age, gender, BMI, and aortic diameter. Within each group, means with means or medians with 95% CIs will be calculated. Following uni- and bivariate analyses and assessment for colinearity, a variety of parametric and nonparametric regression procedures will be conducted. The smoothed curves will be approximated using a modified LMS estimation procedure.

**Results:**

Data for the initial curve derivation includes 25 patients ranging from 13 months to 20 years (mean 102 months or 8.5 years). Sixty-five percent of patients were enrolled from the ED, while 35% were enrolled from well-child clinic visits. When evaluating the size of IVC as a function of time linear growth, increasing size was found to proportionately increase with age of patient in months.

**Conclusions:**

Data suggest a linear correlation between IVC size and age. Such data, when plotted as a new growth curve, may allow clinicians to plot a patient's sonographic measurements in order to assess hydration health.

## Background

Measurement of inferior vena cava (IVC) diameter and its correlation to aortic diameter have received much interest in recent ultrasound research as a means to assess fluid status. Due to age-dependent changes in the diameter of the IVC, several prospective, controlled, observational studies have assessed the vena caval to aortic ratio (CAR) and found a lower ratio in children clinically assessed to be dehydrated. Furthermore, these studies found that the CAR increases sonographically with repeated fluid boluses [1,2]. In adult critical care research, central venous pressure (CVP) analysis and pulmonary artery occlusion pressure (PAOP) have both been studied and used to assess pre- and post-hydration volume status. The CVP is commonly used to identify the patient who is preload or volume sensitive. Yet in children, it is known to be a less accurate predictor [3]. The use of PAOP as a means of guiding volume replacement has also been questioned [4]. Accurate assessment of fluid status is critical in the pediatric patient population; but due to the invasiveness of CVP and PAOP, they are not commonly used in the pediatric population. Rather, clinical signs such as capillary refill, heart rate, and skin turgor coupled with patient history are the leading assessment tools utilized by pediatric clinicians. Use of patient dry weight is often cited as a tool for primary care and inpatient settings for determining hydration status, but this is not easily assessed or compared to baseline in an emergent setting. Use of clinical signs alone was found to lack sensitivity in a sizable pediatric analysis completed by Gorelick et al. [5]. Given the dynamic changes observed in the vasculature pre- and post-hydration, ultrasound analysis of the IVC is emerging as the leading tool in terms of both sensitivity and specificity of hydration state assessment in children.

**Figure 1:**
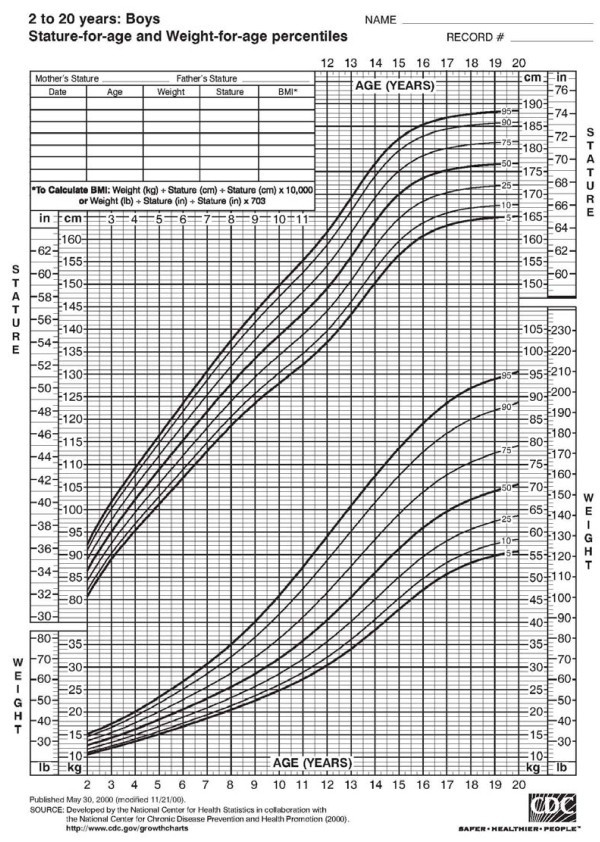
**Standard growth curve for boys (height) [**[6]**].**

Proposed is the derivation and validation of a new growth curve of the IVC as a function of age, body mass index (BMI) and/or caval aortic ratio, comparable to the height and weight curves in current practice (see Figure 1) [6]. The results of the initial investigation of feasibility of curve derivation are presented herein.

## Methods

Children between the ages of 4 weeks and 20 years were eligible for the study. Parents or children were considered for enrollment by the treating clinician at two sites: an urban pediatric emergency department (pediatric census: 20,446) and an urban pediatric outpatient clinic. At the clinic, only patients presenting for well-child visits, and in the ED, only patients evaluated for complaints known not to change hydration state, were screened and enrolled. New York Methodist Hospital institutional review board approved this study. All patients or their legal guardians provided written informed consent.

Once written consent was obtained and the patients enrolled, each underwent a short (<5 min) trans-abdominal ultrasound using one of two machines (Zonare Ultra, Mountain View, CA or Sonosite Turbo*,* Bothell, WA) Patient specific data, other than US measurements, were collected and recorded by treating physicians; and included height and weight to calculate body mass index.

Five US-credentialed clinicians, ranging from post-graduate year (PGY)4 to PGY6 completed a 1-h introduction to IVC-US and ten supervised scans prior to enrollment. Still, images will be measured in order to determine IVCDi in both transverse and longitudinal planes. To assess inter-rater reliability, in 10% of cases, two clinicians will complete scans. All study scans will be over-read by a fellowship-trained sonologist.

Diameter of IVC at minimum and maximum diameter as well as aortic diameter in both longitudinal and transverse planes was digitally recorded by investigators. Measurements were obtained 1 to 2 cm below the level of the hepatic veins using subcostal or subxiphoid windows. Plots were completed using IVC diameter as a function of age and BMI to assess the initial shape of the curve and reassessed power calculations.

### Primary outcome measures

Size (in centimeters) of inferior vena cava and size of aorta were measured at minimum and maximum diameters in both longitudinal and transverse planes in all study patients; as previously described [7,8]. Still, video clips were recorded and reviewed by the ultrasound fellowship director.

### Independent variables

Height, weight, age (in months), and reason for emergency or clinic visit were recorded for all study patients. Using patient height and weight in pounds, BMI was calculated to the nearest whole number. Gender, ethnicity, history of abdominal surgery, and vital signs were also recorded.

### Analysis

In order to assess the relationship of the IVC diameter with patient age, the minimum and maximum measurement of IVC diameters were plotted. CAR was also plotted against age in months. Following uni- and bivariate analyses and assessment for colinearity, a variety of parametric and nonparametric regression procedures will be conducted. The smoothed curves will be approximated using a modified LMS estimation procedure.

## Results and discussion

**Figure 2:**
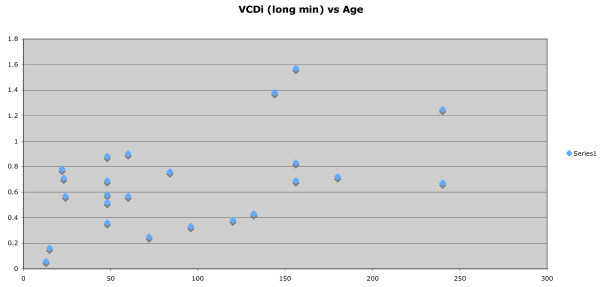
Minimum IVC diameter as a function of age in months.

**Figure 3:**
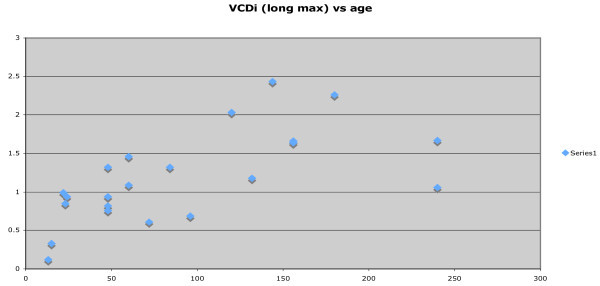
Maximum long IVC diameter as a function of age in months.

**Figure 4:**
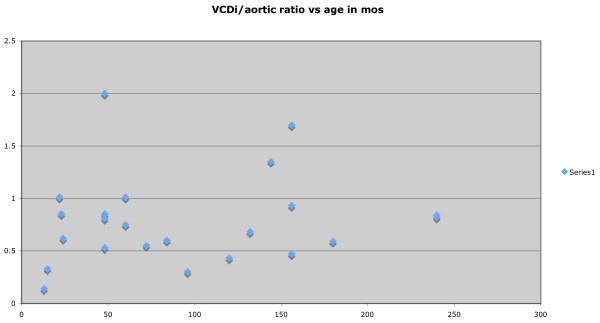
CAR as a function of age in months.

Twenty-five patients were enrolled and imaged. The patients ranged in age from 13 months to 240 months. IVCDi ranged from 0.12 to 2.26 cm across all patients. Mean IVCDi minimum was 0.67 cm, and mean IVCDi maximum was 1.19 cm. The aortic size ranged from 0.7 to 1.65 cm; with a median aortic size of 1.05 cm. CAR ranged from 0.3 to 2 with a median CAR of 0.84. As demonstrated in Figures 2 and 3, the relationship between age and IVCDi has a linear correlation. The most closely correlated data set was that of maximum IVCDi in the long-axis plane versus the patient age in months. (Figure 3). CAR is plotted as a function of age in months in Figure 4 and is also correlated linearly but with a more modest slope.

### Limitations

Given the range of ages examined and plotted with respect to IVCDi, the small number of patients was assessed at the current time limits applicability and accuracy of the curve. As this initial investigation was completed and the analysis of plotted curves progressed, there was a trend toward more linear correlation with increased patient enrollment. With continuation of enrollment, the confidence in the relationship between age and IVCDi should strengthen.

## Conclusions

Sonographic measurement of IVC dimensions can be performed by emergency clinicians with limited experience in both emergency department and outpatient clinic settings. Once the IVC grow curve is validated, clinicians will have a rapid, accessible, and reliable set of age and weight-related sonographic indices for use in the assessment of the hydration status of pediatric patients.

## Competing interests

The authors declare that they have no competing interests.

## Authors' contributions

EJH participated in the study design, data acquisition, data analysis, and manuscript writing. GCC participated in the study design, data acquisition, data analysis, and manuscript writing and editing. KA participated in data acquisition. WMB participated in data analysis. RVA participated in manuscript revision. AL and KOR participated in data acquisition. LM participated in study design, data analysis, and manuscript revision. All authors read and approved the final manuscript.

## Authors' information

EJH is a doctor of medicine and an attending Pediatric Emergency Medicine physician at New York Methodist Hospital in Brooklyn, New York. GCC is a doctor of medicine, a member of the FACEP, and the chair of the division of point of care ultrasound and an attending Emergency Medicine physician at New York Methodist Hospital in Brooklyn, New York. KA is the doctor of medicine and a pediatric resident physician at New York Methodist Hospital in Brooklyn, New York. WMB was a biostatistician at New York Methodist Hospital in Brooklyn, New York. RVA is the doctor of medicine and the chair of pediatric emergency medicine at New York Methodist Hospital in Brooklyn, New York. KOR is the doctor of medicine and the assistant director of emergency ultrasound and an attending Emergency Medicine physician at New York Methodist Hospital in Brooklyn, New York. AL is the doctor of medicine and an emergency ultrasound fellow at New York Methodist Hospital in Brooklyn, New York. LM is the doctor of medicine and vice chair and attending physician of Emergency Medicine at New York Methodist Hospital in Brooklyn, New York.
